# Electronic Clinical Quality Measures of Antibiotic Use in Hospitalized Adults With Uncomplicated Community-Acquired Pneumonia

**DOI:** 10.1093/cid/ciag273

**Published:** 2026-04-21

**Authors:** Andrea T White, Matthew Bidwell Goetz, Makoto Jones, Melinda M Neuhauser, Claire Ciarkowski, David Ratz, Ashwin Gupta, Lindsay A Petty, Tejal N Gandhi, Mingyuan Zhang, Brittany Kent, Chaorong Wu, Jennifer K Horowitz, Scott A Flanders, Arjun Srinivasan, Valerie M Vaughn

**Affiliations:** Division of General Internal Medicine, Department of Internal Medicine, University of Utah School of Medicine, Salt Lake City, Utah, USA; Infectious Diseases Section, VA Greater Los Angeles Healthcare System, David Geffen School of Medicine at UCLA, Los Angeles, California, USA; Medicine Service, Infectious Diseases Section, VA Salt Lake City Healthcare System, Salt Lake City, Utah, USA; Division of Healthcare Quality Promotion, Centers for Disease Control and Prevention, Atlanta, Georgia, USA; Division of General Internal Medicine, Department of Internal Medicine, University of Utah School of Medicine, Salt Lake City, Utah, USA; Center for Clinical Management Research, Veterans Affairs Ann Arbor Health System, Ann Arbor, Michigan, USA; Division of Hospital Medicine, Department of Internal Medicine, University of Michigan Medical School, Ann Arbor, Michigan, USA; Medicine Service, Veterans Affairs Ann Arbor Healthcare System, Ann Arbor, Michigan, USA; Division of Infectious Diseases, Department of Medicine, University of Michigan Medical School, Ann Arbor, Michigan, USA; Division of Infectious Diseases, Department of Medicine, University of Michigan Medical School, Ann Arbor, Michigan, USA; Data Science Services, University of Utah, Salt Lake City, Utah, USA; Division of Epidemiology, Department of Internal Medicine, University of Utah, Salt Lake City, Utah, USA; Division of General Internal Medicine, Department of Internal Medicine, University of Utah School of Medicine, Salt Lake City, Utah, USA; Division of Hospital Medicine, Department of Internal Medicine, University of Michigan Medical School, Ann Arbor, Michigan, USA; Division of Hospital Medicine, Department of Internal Medicine, University of Michigan Medical School, Ann Arbor, Michigan, USA; Division of Healthcare Quality Promotion, Centers for Disease Control and Prevention, Atlanta, Georgia, USA; Division of General Internal Medicine, Department of Internal Medicine, University of Utah School of Medicine, Salt Lake City, Utah, USA; Medicine Service, Infectious Diseases Section, VA Salt Lake City Healthcare System, Salt Lake City, Utah, USA; Division of Hospital Medicine, Department of Internal Medicine, University of Michigan Medical School, Ann Arbor, Michigan, USA

**Keywords:** antibiotic duration, empiric antibiotic therapy, antimicrobial stewardship, patient safety measures

## Abstract

**Background:**

Community-acquired pneumonia (CAP) is the top indication for inpatient antibiotic use and overuse. We describe the development of 2 electronic clinical quality measures (eCQMs) assessing the percentage of hospitalized patients with uncomplicated CAP who had unsupported antibiotic duration beyond 5 days and empiric antibiotics targeting methicillin-resistant *Staphylococcus aureus* and/or *Pseudomonas aeruginosa*.

**Methods:**

Both eCQMs were developed based on clinical guidelines, literature, and expert feedback and then specified electronically. The eCQMs were applied to patient data from 2 academic medical centers and 109 Department of Veterans Affairs (VA) healthcare systems to evaluate importance, reliability, validity, and feasibility, as defined by the Centers for Medicare and Medicaid Services.

**Results:**

Across all hospitals, the mean percentage of eligible patients with unsupported excess duration and inappropriate empiric antibiotic use ranged from 40% to 55% and from 17% to 56%, respectively. Within the VA cohort, reliability (median [first−tenth decile, n]) for duration and empiric eCQMs was high: 93% (63%–97%, 28 238) and 91% (63%–96%, 47 034), respectively. When compared to chart review at 1 academic hospital, both eCQMs had high sensitivity (96%, both) and specificity (92% and 93% for duration and empiric eCQMs, respectively). Feasibility was maximized by using only data elements in structured electronic health record fields. Some technically complex elements (eg, clinical stability) were retained for face validity.

**Conclusions:**

Two eCQMs identifying unsupported excess antibiotic duration and inappropriately broad empiric antibiotic use in patients with uncomplicated CAP had high importance, reliability, validity, and feasibility. These eCQMs could be benchmarked to assist antibiotic stewardship programs in understanding and improving their antibiotic use.

## Teaser

We developed 2 electronic quality measures for antibiotic duration and empiric selection in hospitalized patients with uncomplicated community-acquired pneumonia. Both measures had high reliability (93% and 91%, respectively), sensitivity (96%, both measures), and specificity (92% and 93%, respectively).

Any antibiotic use can lead to adverse events and antibiotic resistance, but antibiotic overuse puts patients at risk without benefit [1–10]. Pneumonia has been shown to be a top reason for antibiotic prescribing in all acute care hospitals, accounting for roughly one-third of all hospital antibiotics used for infections [11]. For community-acquired pneumonia (CAP), shorter antibiotic durations may be associated with fewer antibiotic-associated adverse events and less risk of developing antibiotic-resistant infections [2, 4, 12]. An analysis of a multihospital cohort found that for each additional day of excess antibiotic use, 30-day patient-reported antibiotic-associated adverse events were more likely (adjusted odds ratio for each excess day, 1.03 [1.00–1.06]) [4] and that reducing excess duration may reduce antibiotic-associated adverse events [13]. Similarly, unnecessarily broad empiric therapy is associated with adverse consequences, including increased risk of death, kidney injury, and serious secondary infections [14–16].

To avoid harms from antibiotic overuse, national organizations, including the Centers for Disease Control and Prevention [17], the Joint Commission [18], and the Centers for Medicare and Medicaid Services (CMS) [19], have made improving antibiotic use for CAP a priority. Generally, disseminated measures of antibiotic use have not focused on including the granular clinical details necessary to approximate clinical antibiotic appropriateness. This is due, in part, to a lack of availability and standardization of patient-level data. Greater implementation of electronic health records (EHRs) and movements toward data standardization (eg, through Fast Healthcare Interoperability Resources) now make measures of antibiotic appropriateness more feasible.

Here, we describe the development, final characteristics, and testing of 2 electronic clinical quality measures (eCQMs) to identify unsupported excess antibiotic duration and inappropriately broad empiric therapy. The eCQMs complement existing antibiotic quality measures (eg, those in the National Healthcare Safety Network Antimicrobial Use Option) [20] by providing concrete feedback data related to uncomplicated CAP. Both eCQMs were conceptually based on chart review measures that assessed antibiotic duration and selection, which were shown to reduce antibiotic overuse and antibiotic-associated events in a cohort of 41 Michigan hospitals [4, 13]. Both eCQMs received endorsement with conditions following a national, consensus-based review facilitated by the Partnership for Quality Measurement (PQM), a CMS-certified entity led by Battelle, confirming they met CMS criteria for importance, validity, reliability, and feasibility.

## METHODS

**Figure 1.**
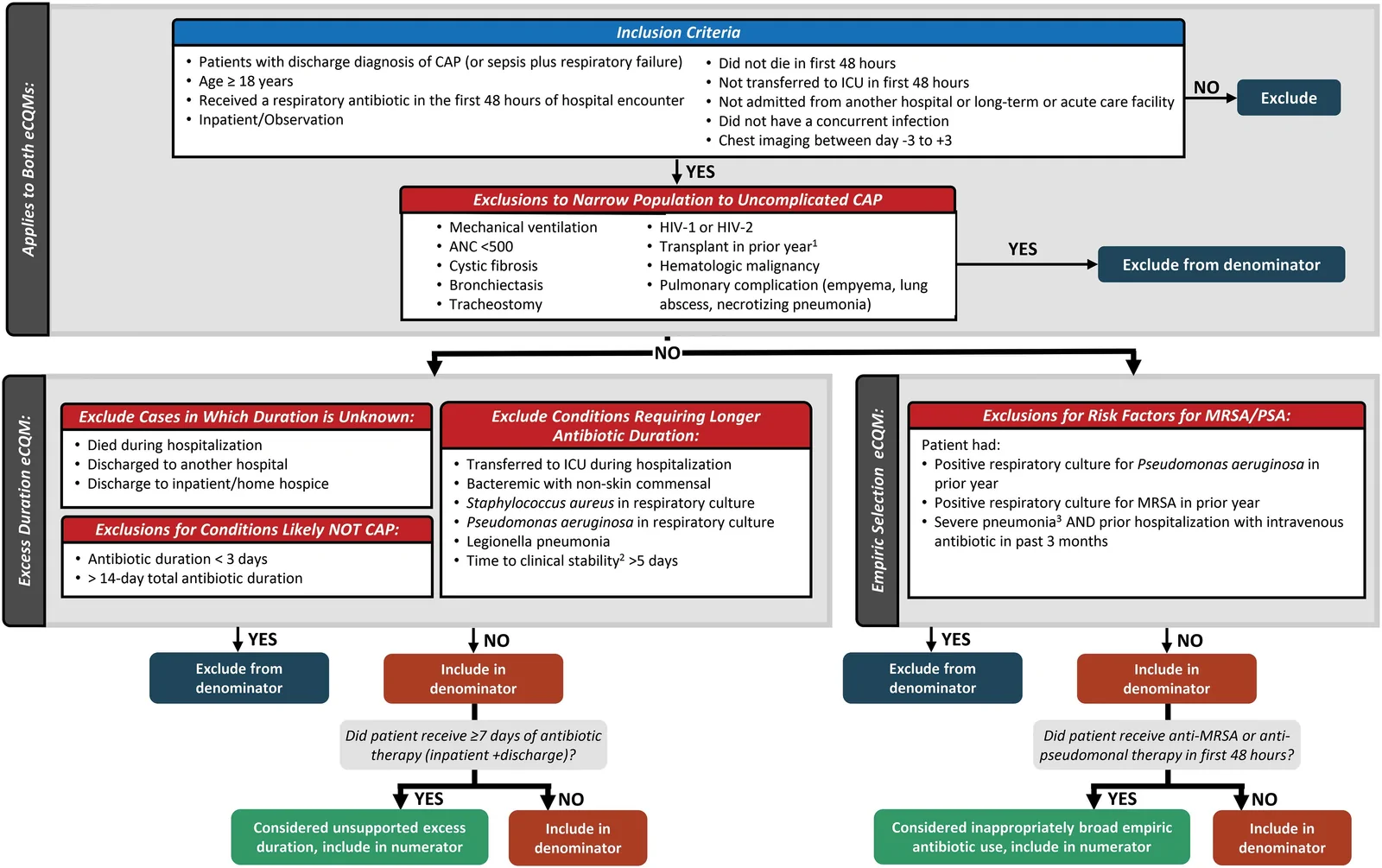
Measure specifications and flow chart to determine eCQM scores for excess antibiotic duration and inappropriately broad empiric antibiotic therapy eCQMs. ^1^Any transplant code in prior year. Meant to capture lifetime transplant history. ^2^Modified definition is afebrile (temperature ≤37.8°C) and normotensive (systolic blood pressure [SBP] >90 mm Hg) OR discharged. ^3^Modified definition is 2 or more of the following on a single day in the first 48 hours of the hospital encounter: respiratory rate >30 breaths/min, blood urea nitrogen >20 mg/dL, leukocyte count <4000 cells/mL, platelets <100 000/mL, temperature <36.0°C, SBP <80 mm Hg. Abbreviations: ANC, absolute neutrophil count; CAP, community-acquired pneumonia; eCQM, electronic clinical quality measure; HIV, human immunodeficiency virus; ICU, intensive care unit; MRSA, methicillin-resistant *Staphylococcus aureus.*

We developed 2 adult eCQMs that used structured data from the EHR to quantify the percentage of hospitalized patients with uncomplicated CAP who had guideline-discordant antibiotic duration that exceeded 5 days [21] and empiric antibiotic therapy that targeted methicillin-resistant *Staphylococcus aureus* (MRSA) and/or *Pseudomonas aeruginosa* [22]. Patients were eligible for inclusion if they were adults (aged ≥18 years) hospitalized with CAP (defined by discharge diagnosis plus receipt of chest imaging and respiratory antibiotic; see Supplementary Table 15 for list) who did not have a concurrent infection. From this population, “uncomplicated” CAP was identified by excluding patients with electronically identifiable severe immune suppression, severe underlying lung disease, or a pulmonary complication (see Figure 1 for full inclusion and exclusion criteria).

Once patients with uncomplicated CAP were identified, we then excluded patients if a duration >5 days or empiric treatment with anti-MRSA or anti-pseudomonal antibiotics could be considered appropriate or supported. While there are different nomenclatures for describing antibiotic overuse, we use “unsupported” (ie, “use of antimicrobials that deviated from recommended guidelines”) [23] to describe both eCQMs; “excess duration” (ie, “antibiotics prescribed beyond the indicated duration of therapy, absent any clinical reason for a lengthened course”) [24] for a duration >5 days; and “inappropriate” (ie, “use of antimicrobials not recommended in treatment guidelines”) [25] to describe empiric treatment with anti-MRSA or anti-pseudomonal antibiotics without an indication for broad-spectrum coverage.

For the duration assessment, we excluded patients with electronically identifiable reasons for prolonged therapy, per the 2019 Infectious Diseases Society of America (IDSA)/American Thoracic Society (ATS) CAP guidelines [26], including those that were bacteremic; had *Legionella*, *S. aureus*, or *P. aeruginosa* infection; were transferred to the intensive care unit during hospitalization; or were not clinically stable (nor discharged) by hospital day 5. We defined clinically stable as afebrile, normotensive, or discharged (see Feasibility section later in this section for definition details). To account for difficulty counting half days in antibiotic prescriptions, we operationalized excess duration in the eCQM as receiving ≥7 days of total (inpatient plus discharge) antibiotic therapy; so as not to imply we are recommending a 7-day duration, we refer to excess duration as >5 days.

For the empiric assessment, we excluded patients with electronically identifiable risk factors for MRSA or *P. aeruginosa* as specified in the 2019 IDSA/ATS CAP guidelines (ie, positive respiratory culture for MRSA/*P. aeruginosa* in prior year or hospitalization within the last 90 days with receipt of intravenous antibiotics) [26, 27]. Originally, we anticipated using the empiric measure to assess empiric antibiotic therapy for all hospitalized patients, with categories of “underuse” included. However, 7 of 8 members of our Technical Expert Panel (TEP, described in Data Sources below) recommended that the eCQM focus on potential MRSA/*P. aeruginosa* therapy overuse.

See full inclusion and exclusion criteria for both eCQMs in Figure 1 (further details in pages 2–5 of the Supplementary Material).

### Data Sources

Both measures were developed using data from 4 primary sources: the University of Michigan (UM), University of Utah (UU), the Department of Veterans Affairs (VA) healthcare systems (ie, 1 or more VA medical centers that share an EHR instance), and 2 TEPs.

#### UM: Epic and Michigan Hospital Medicine Safety Consortium

eCQM specifications were applied to UM's Electronic Data Warehouse to obtain measure data from CAP hospitalizations between 29 September 2015 and 11 December 2021. For validity testing, we compared UM's eCQM-derived data to matching cases with chart review data collected by the Michigan Hospital Medicine Safety (HMS) Consortium. HMS, a statewide collaborative quality initiative, manually collects all data necessary to assess both measures (including denominator/numerator inclusions and exclusions) and has used chart review–based versions of both measures to improve care for CAP since 2015 [13, 24, 28, 29]. Quality assurance data for HMS have been published previously [13, 24, 28, 29], and HMS data have been used to inform prior quality measures (Consensus-Based Entity [CBE] identification [ID] 3671 and 3690) [30–32].

#### UU: Epic

To ensure the eCQM could be assessed in differing Epic instances, we also obtained eCQM data from the UU's Electronic Data Warehouse on CAP hospitalizations between 1 January 2021 and 30 May 2022.

#### Veterans Health Information Systems and Technology EHR

eCQM specifications were applied to data derived from VA's Veterans Health Information Systems and Technology (VistA) EHR, including 109 VA healthcare systems, on all hospitalizations for CAP between 1 January 2022 and 30 June 2024. Similar to prior quality measures (CBE ID: 4440e) [33], we used the VA data to assess reliability and variation across hospitals.

#### TEPs

We held 2 TEPs (1 for each measure) comprised of stakeholders with diverse perspectives and areas of expertise who work in a variety of professional, academic, and nonprofit settings (Supplementary Table 1). The TEPs provided expert feedback to help inform measure specifications during two 2-hour sessions held in April 2023 and via a post-session online questionnaire.

### CMS’ Measure Endorsement Criteria

CMS’ consensus-based entity, currently awarded to Battelle's PQM, initially endorses national quality measures based on 3 criteria [34]: importance, scientific acceptability (eg, reliability, validity), and feasibility. Additional criteria of use/usability and closing care gaps (ensuring high-quality care for all) are required for maintenance of endorsement but not for initial endorsement and thus are not discussed here. CMS definitions of criteria and a summary of data sources we used to assess each of these are presented in Table 1 and described below.

**Table 1.** Measure Endorsement Criteria, as Defined by the Centers for Medicare and Medicaid Services, and Description of Data Sources and Tests Performed

Measure Domains	Our Data Source(s) and Methods
*Importance:* Extent to which the measure is important for making significant gains in healthcare quality or cost where there is variation in or overall less-than-optimal performance	Support for variation in scores across hospitals VA VistA data from 109 VA healthcare systems^a^ including eligible hospitalizations (n = 47 034 and n = 28 238 for duration and empiric eCQMs) between 1 January 2022 and 30 June 2024, we computed scores by decile to assess score gaps.Epic data from University of Utah between 1 January 2021 and 30 May 2022 and University of Michigan between 29 September 2015 and 11 December 2021
*Scientific acceptability:* Reliability: Extent to which the measure is well defined and precisely specified so it can be implemented consistently within and across organizations and allows for comparability	*Accountable entity-level reliability* Signal-to-noise analysis and intraclass correlation of VA data across 109 healthcare systems (between 1 January 2022 and 30 June 2024) to determine: Reliability for the entire hospital cohort using median number of eligible cases Minimum number of annual cases needed to achieve reliability thresholds of 0.6, 0.7, 0.8, 0.9 Mean reliability score by decile of reliability calculated using beta-binomial regression
*Scientific acceptability:* Validity: Extent to which the measure data elements are correct and the measure scope correctly reflects the quality of care provided, adequately identifying differences in quality	*Face validity* Measure definitions and exclusions adhered to American Thoracic Society /Infectious Disease Society of America clinical practice guidelines unless otherwise stated [27, 28] Assessed via national TEP feedback *Encounter-level validity* Comparison of eCQM results with case review results to assess validity (sensitivity, specificity) of critical data elements (eg, exclusion criteria) Comparison of eCQM results with case review results for measure assessment (eg, excess duration vs not) to determine overall measure sensitivity and specificity (also report percent agreement) *Empirical Validity* Association of the 2 eCQMs at the hospital level (ie, do hospitals that perform better on one eCQM, perform better on the other?)
*Feasibility:* Extent to which the measure specifications (ie, numerator, denominator, exclusions) require data that are readily available OR could be captured without undue burden AND can be implemented for performance measurement	Confirming that all elements of the eCQMs are available in the EHR. Measure data elements were made more feasible based off TEP suggestions. Final data element availability confirmed for the following EHRs:
	Epic: University of Michigan and University of Utah
	VistA: Veterans Affairs healthcare systems

^a^VA “healthcare systems” refers to 1 or more VA medical centers that share an EHR instance.

Abbreviations: eCQM, electronic clinical quality measure; EHR, electronic health record; TEP, Technical Expert Panel; VA, Department of Veterans Affairs; VistA, veterans health information systems and technology electronic health record.

#### Importance

A key element of importance is a demonstrated care gap or variation in care. In addition to published evidence of variation in care [1, 3, 4, 15, 35, 36], we compiled eCQM scores by decile for the 109 VA healthcare systems. We also report UU and UM scores.

#### Scientific Acceptability: Reliability

Per PQM, reliability is the extent that the measure can be implemented consistently within and across organizations. We conducted reliability testing with 2 models using data from the 109 VA healthcare systems described above. The first was a signal-to-noise analysis using a mixed-effect logistic model, enabling calculation of the hospital variance (signal), total variance, residual variance (noise), and the intraclass correlation (ICC). Using the Spearman–Brown formula, we then used the ICC to calculate the reliability for the entire hospital cohort using the median number of cases for the cohort and to determine the minimum number of cases necessary to achieve prespecified reliability thresholds (0.6, 0.7, 0.8, and 0.9). Finally, we sorted the VA healthcare systems by decile of reliability (after exclusions) and calculated the mean reliability score for each decile using beta-binomial regression (using SAS code provided in *The Reliability of Provider Profiling: A Tutorial*) [37].

#### Scientific Acceptability: Validity

For both eCQMs, we assessed face validity, 2 types of encounter-level validity (how well data elements or an encounter-level construct compares to a reference standard), and empirical validity (the extent to which the measure reflects quality of care). In the PQM submission, we also noted previously published empirical validity results [1, 2, 4, 8, 12, 15, 36].

##### Face Validity

Both eCQMs were based on national guidelines for CAP; literature reviews that included randomized; clinical trials; measure use within HMS and the VA; and feedback and review from our TEP. In our TEP questionnaires, we asked each expert to assess how well the measures could distinguish between better and worse performing hospitals and if there were any key data elements that were missed or not appropriately used in each measure.

##### Encounter-Level Validity: Comparison With Case Review

We compared data from UM patients who had both electronic (from the Enterprise Data Warehouse) and chart review (from HMS) data for appropriateness assessments. Results were reported as percentage agreement for antibiotic duration (total, inpatient, and discharge days) and categories of empiric antibiotics (ie, no anti-MRSA/pseudomonal, only anti-MRSA, only anti-pseudomonal, or both anti-MRSA/pseudomonal) and sensitivity/specificity for identifying excess antibiotic duration and inappropriately broad empiric therapy.

##### Encounter-Level Validity: Critical Data Element Validity

Similarly, we compared critical data elements (eg, exclusion criteria) between UM eCQM data and HMS chart review data (reference standard) to determine sensitivity and specificity. For exclusions not available in the existing chart review database, we compared eCQM exclusion rates across the 3 health systems.

##### Empirical Validity

To determine if performance on one measure was related to performance on the other, we used data from the 109 VA healthcare systems to assess the association (at the hospital level) between excess antibiotic duration for CAP and guideline-discordant anti-MRSA/anti-pseudomonal empiric antibiotics for CAP.

#### Feasibility

For both eCQMs, a major goal was to leverage the most reliable, accurate, and standardized data elements to provide a simple, transparent measure that could be consistently extracted, calculated, and interpreted. We tested both eCQMs within 3 health systems to determine whether all required data elements were routinely generated during the care of hospitalized patients with CAP, which barriers or challenges exist when implementing and extracting the measure from current healthcare data to inform a “feasibility scorecard” [21, 22] (ie, the extent that measure elements were accessible in structured data), and whether any elements were uncommon or able to be removed to simplify the measure (ie, their removal had no impact on measure performance and limited impact on face validity). Feasibility was also conceptually considered in conversations with the TEP who provided input on when to prioritize face validity over feasibility (or vice versa).

#### Ethics Approval

Elements of these projects were approved by the VA Greater Los Angeles Healthcare System, the University of Utah, and the University of Michigan Health System institutional review boards. (See 45 C.F.R. part 46.101(c); 21 C.F.R. part 56).

## RESULTS

### Importance

**Figure 2.**
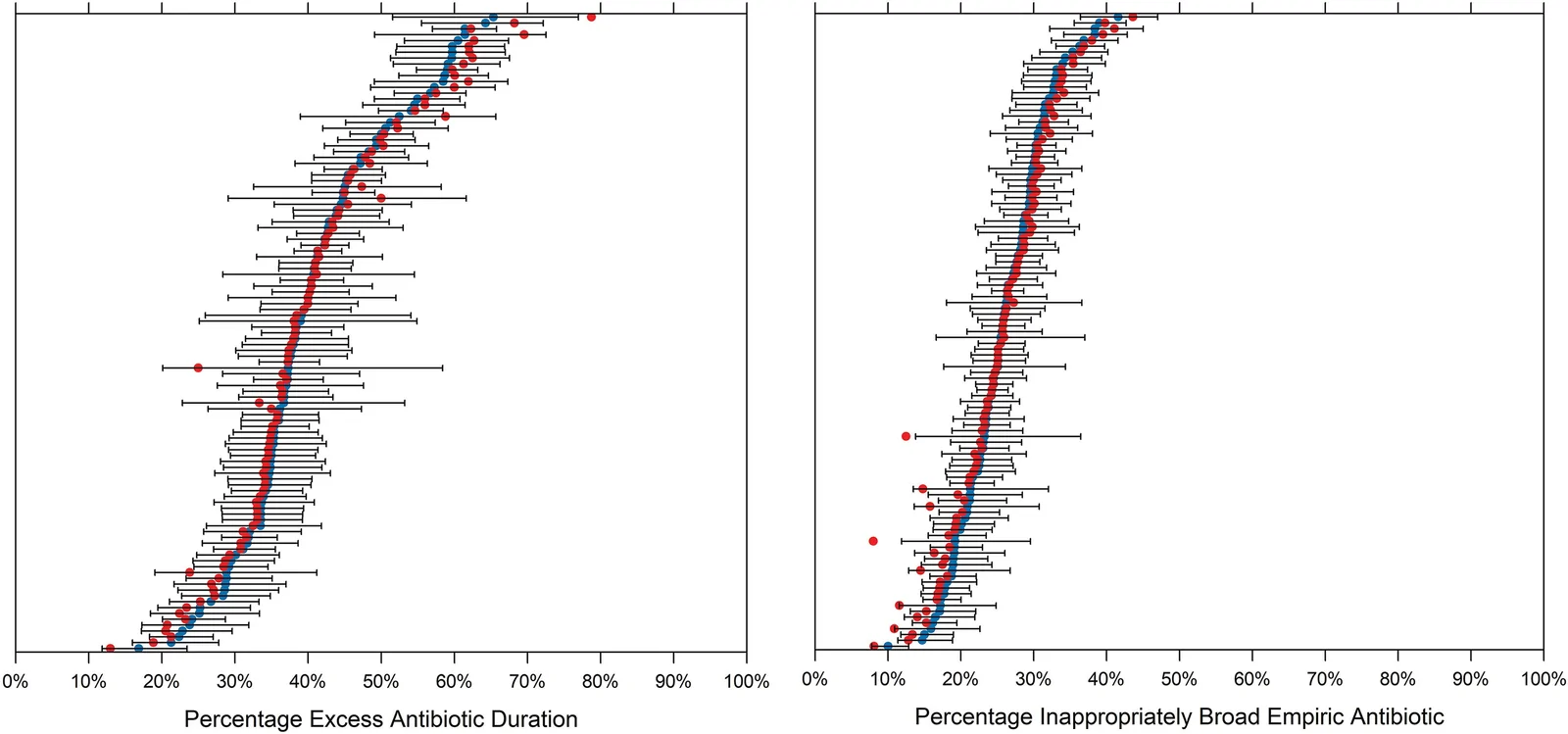
Percentage of patients hospitalized with community acquired pneumonia (CAP) who received excess antibiotic duration (as defined by our electronic clinical quality measure) (*A*) or inappropriately broad empiric antibiotic therapy (*B*) across 109 Department of Veterans Affairs healthcare systems (1 January 2022–30 June 2024). Each row of the caterpillar plot represents a single VA healthcare system with the raw (unadjusted) percentage in red and the estimated percentage from a mixed-effects logistic model with healthcare system random effects shown in blue (the bar represents the 95% confidence interval). Model-predicted estimates are shrunk toward the mean, meaning that the smaller the number of samples from a particular healthcare system, the more the estimate for that system is pulled toward the global mean.

We found large variation in scores when we applied the eCQMs to our 3 electronic data cohorts: the mean percentage of hospitalized patients with uncomplicated CAP who had unsupported excess duration and inappropriately broad empiric antibiotic use ranged from 39.8% to 54.5% and from 16.6% to 55.9%, respectively (Supplementary Table 2). Within the cohort of 109 VA healthcare systems (grouped by deciles), measure scores also varied, with 33.4%–46.2% (28 238 patients) of hospitalized patients with excess duration and 19.2%–32.7% (47 034 patients) with inappropriately broad empiric therapy (see Figure 2 and Supplementary Tables 3 and 4).

### Scientific Acceptability

#### Accountable Entity-Level Reliability

Across 109 VA healthcare systems, 84.4% and 86.2% of systems had the minimum number of cases (n = 19 and n = 56) necessary to achieve 0.70 reliability for duration and empiric measures, respectively, supporting that most hospitals would have sufficient cases to achieve acceptable reliability (Supplementary Table 5). For the duration and empiric measures, VA healthcare systems with the median number of eligible cases (n = 49 and n = 135, respectively) had a reliability of 80.1% and 85.0%, respectively.

Based on our signal-to-noise analysis, overall reliability for the first and tenth decile hospitals was 62.7%–97.3% for duration and 62.5%–96.3% for empiric measures (complete accountable entity-level reliability testing in Supplementary Tables 3 and 4).

#### Validity

Table 2 summarizes validity testing results.

**Table 2.** Source Data and Results of Validity Testing

Type of Validity Testing (Source Data)	Results
*Face validity*	
Face validity, TEP: TEP response to: “The measures can distinguish between better and worse performing hospitals” and “Are there any key data elements you believe are missed or not appropriately used?”	Duration eCQM: 8/8 (100%) agreed/strongly agreed Empiric eCQM: 5/7 (71%) agreed/strongly agreed Duration: No Empiric: “The clinical stability definition is … complex and I am concerned that the initial pilot results will not play out … due to outlier vital signs values … and data completeness…” → In response, we simplified the definition of clinical stability for the measure (see discussion in Feasibility section)
Encounter-level validity:	
Comparison with chart review, percent agreement: n = 450 (duration) and n = 540 (empiric) cases	Duration eCQM: 91%–97% agreement within ±1/2 d duration between eCQM and chart review for total, inpatient, and discharge antibiotic durations; 95%–99% agreement within ±1.5 d (Supplementary Tables 6–8) Empiric eCQM: eCQM accurately assessed empiric antibiotic classifications (no anti-MRSA/pseudomonal, only anti-MRSA, only anti-pseudomonal, and both) in 97% of patients (Supplementary Table 9)
Comparison with chart review, sensitivity and specificity: n = 450 (duration) and n = 540 (empiric)	Duration eCQM: UM eCQM found 61.4% of cases (n = 281) received unsupported excess duration; UM chart review found 60.7% (n = 278); sensitivity = 96%, specificity = 93% Empiric eCQM: UM eCQM found 53.0% (n = 286) had unsupported inappropriately broad empiric antibiotic therapy; UM chart review found 54.4% (n = 294); sensitivity = 96%, specificity = 92%
Validity of exclusion criteria: n = 592 hospitalized patients with community acquired pneumonia	Duration eCQM: Specificity of exclusion criteria ranged from 98% to 100%; sensitivity ranged from 88% to 100% with “time to clinical stability” as an outlier with a sensitivity of 29% Empiric eCQM: Specificity of exclusion criteria ranged from 78% to 99%; sensitivity ranged from 60% to 100%
Empirical validity	
Hospital-level association between duration and empiric therapy measure performance (VA data from 109 healthcare systems)	Measures were mildly correlated with each other at the hospital level (R = 0.3, *P* = .001)

Abbreviations: eCQM, electronic clinical quality measure; MRSA, methicillin-resistant *Staphylococcus aureus;* TEP, Technical Expert Panel; UM, University of Michigan; VA, Department of Veterans Affairs.

##### Face Validity

When the TEPs were asked if measures could distinguish between better and worse performing hospitals, 100% (8 of 8, duration) and 71% (5 of 7, empiric) agreed or strongly agreed (survey results in Supplementary Material).

##### Encounter-Level Validity: Comparison With Chart Review

For antibiotic duration (ie, total, inpatient, and discharge durations), agreement between eCQM and chart review data was high: 91%–97% within ± ½ day and 95%–99% within 1.5 days (Table 2, details in Supplementary Tables 6–8). For empiric antibiotic therapy, agreement between eCQM and chart review data was highest for patients who received neither anti-MRSA nor anti-pseudomonal treatment or who received both (99.5% and 97.5%, respectively; Supplementary Table 9). Similarly, when compared to chart review, both eCQMs accurately identified unsupported antibiotic use (Table 2).

##### Encounter-Level Validity: Validity of Exclusion Criteria

The sensitivity and specificity of individual exclusion criteria when comparing UM electronic data to HMS chart review are shown in Table 3. Generally, sensitivity and specificity were high, except for the definition of clinical stability (which we purposely made less sensitive based on TEP feedback to increase feasibility; see more in the Feasibility section). eCQM exclusion rates were similar across the 3 EHRs (Supplementary Tables 10–13).

**Table 3.** Prevalence and Sensitivity/Specificity of Electronic Clinical Quality Measure (eCQM)–Specific Exclusion Criteria Comparing eCQM and Chart Review

Exclusion	UM ECQM Data (electronic), N (%)	UM HMS Data (chart Review), N (%)	Sensitivity of UM ECQM (vs UM HMS Chart Review)	Specificity of UM ECQM (vs UM HMS Chart Review)
Duration-specific exclusions, N = 450 patients				
Died during hospitalization	2 (0.3%)	2 (0.3%)	100% (2/2)	100% (590/590)
Discharged to another hospital	2 (0.3%)	1 (0.2%)	100% (1/1)	99% (590/591)
Discharged to inpatient/home hospice	3 (0.5%)	2 (0.3%)	100% (2/2)	99% (589/590)
Transferred to intensive care unit during hospitalization	9 (1.5%)	9 (1.5%)	88.9% (8/9)	99% (582/583)
Bacteremic with nonskin commensal	11 (1.9%)	8 (1.4%)	88% (7/8)	99% (580/584)
*Staphylococcus aureus* grew from respiratory culture during hospitalization	18 (3.0%)	17 (2.9%)	100% (17/17)	99% (574/575)
*Pseudomonas aeruginosa* grew from respiratory culture during hospitalization	14 (2.4%)	13 (2.2%)	100% (13/13)	99% (578/579)
*Legionella* pneumonia	4 (0.7%)	4 (0.7%)	100% (4/4)	100% (588/588)
Time to clinical stability >5 d^a^	37 (6.3%)	93 (15.7%)	29% (27/93)	98% (489/499)
Received <3 d of antibiotics	35 (5.9%)	31 (5.2%)	100% (31/31)	99% (557/561)
>14-d total antibiotic duration	25 (4.2%)	24 (4.1%)	88% (21/24)	99% (564/568)
Empiric-specific exclusions, N = 540 patients				
Positive respiratory culture for *P. aeruginosa* in prior year	10 (1.7%)	8 (1.4%)	75.% (6/8)	99% (580/584)
Positive respiratory culture for methicillin-resistant *Staphylococcus aureus* in prior year	9 (1.5%)	4 (0.7%)	100% (4/4)	99% (583/588)
Both severe pneumonia^b^ AND prior hospitalization with IV antibiotic in prior 3 m	43 (7.3%)	30 (5.1%)	60% (18/30)	96% (537/562)
Prior admission with IV antibiotics (alone)	101 (17.1%)	114 (19.3%)	70% (80/114)	96% (457/478)
Severe pneumonia (alone)	210 (35.5%)	122 (20.6%)	87% (106/122)	78% (366/470)

Abbreviations: eCQM, electronic clinical quality measure; HMS, Michigan Hospital Medicine Safety Consortium; IV, intravenous; UM, University of Michigan.

^a^Here, we compare the eCQM's simplified definition of clinical stability (afebrile and systolic blood pressure ≥90 mm Hg or discharged) to the original definition of stability (no more than 1 sign of clinical stability). Though the sensitivity is much lower, the feasibility of this specification was much higher across all 3 systems (see Feasibility section for more details).

^b^Here, we compare the eCQM's modified severity criteria (≥2 minor criteria on a single day in the first 48 hours of encounter) to the original American Thoracic Society criteria, which required 1 major or ≥3 minor criteria. The modified severity criteria are much more feasible for the 3 systems the eCQM was tested within (see Feasibility section for more details).

##### Empirical Validity

Within the VA cohort of 109 healthcare systems, the antibiotic duration and broad empiric antibiotic eCQMs were mildly correlated (R = 0.3, *P* = .001; see Supplementary Figure 1).

### Feasibility

Feasibility assessments confirmed that all data elements were available in the EHRs (see feasibility scorecard on PQM site for details) [21, 22].

Specific to the duration measure, we conducted feasibility testing for the definition of “clinical stability.” With TEP feedback, this led to operationalizing stability as “afebrile and normotensive or discharged,” a simpler version than that used in clinical guidelines (temperature ≤37.8°C, heart rate ≤100 beats/min, respiratory rate ≤24 breaths/min, systolic blood pressure ≥90 mm Hg, arterial oxygen saturation ≥90% or pO_2_ ≥60 mm Hg on room air) [26, 38]. This modified definition was feasible for all 3 sites and was highly specific (98% specificity). Notably, the modified definition did not identify all patients who (per chart review) were unstable beyond day 5 (sensitivity 29%). To determine whether the low sensitivity impacted overall measure validity, we compared both definitions within the UM HMS cohort. The original, expanded vital sign criteria resulted in more patients being excluded and 53.7% (1734 of 3227) of patients being quantified as having unsupported excess duration. The more feasible definition resulted in 54.5% (1861 of 3417) of patients being quantified as having unsupported excess duration. Given that the 2 definitions produced comparable results and did not change UM's decile of performance, we used the more feasible, modified stability criteria.

In development of the empiric eCQM, 7 of 8 members of the TEP recommended we remove the proposed “underuse” category and assess only for MRSA/pseudomonal therapy overuse to improve feasibility and understandability of the eCQM. Specific to the empiric eCQM, we conducted feasibility testing for the definition of severe pneumonia. This led to use of a simpler definition of severe pneumonia (≥2 of the following: respiratory rate ≥30 breaths/min, ≥5L oxygen or SpO_2_ <90, blood urea nitrogen ≥20 mg/dL, white blood cell count <4000 cells/uL, platelets <100 000/µL, temperature <36°C, systolic blood pressure <80 mm Hg) than that used in CAP guidelines [26]. When tested in the UM dataset, the modified severity definition had sensitivity of 87% and specificity of 78%. The impact of these modifications is described in Supplementary Table 14, along with other criteria we explored but did not include in the measure definitions due to feasibility concerns.

## DISCUSSION

We developed and tested 2 eCQMs to identify the percentage of adults hospitalized with uncomplicated CAP who have potentially unsupported excess duration and inappropriately broad empiric antibiotic use. After attempting to address TEP feedback for feasibility, the final versions of these eCQMs demonstrated high importance, reliability, validity, and feasibility. In 2025, both eCQMs were endorsed, with conditions, by CMS’ consensus-based entity.

Both eCQMs can feasibly assess antibiotic use electronically and thus could complement other measures, including balancing measures (eg, mortality and readmission), to improve treatment for uncomplicated CAP. Improving adherence to CAP guidelines for antibiotic duration and empiric therapy could help reduce antibiotic-associated adverse events [13] among the roughly 1 million patients hospitalized for pneumonia each year [39] and, theoretically, help address antibiotic resistance.

These eCQMs have limitations. First, they were tested in 2 hospitals with Epic EHRs and the VA VistA. Further testing in other EHRs and in smaller hospitals with fewer information technology (IT) resources is needed to identify issues that affect feasibility, validity, and portability. Second, the eCQM exclusions are extensive. This trade-off was made to improve face validity; however, as part of maintaining endorsement, our group plans to further examine how to simplify the measures, including reducing exclusion criteria, without compromising validity. Third, eCQMs may not be feasible in certain circumstances. For example, critical access hospitals without IT resources may have better success using manual chart review versions. Fourth, we modified some definitions (eg, severity, clinical stability) to improve feasibility, and this decreased sensitivity. Finally, we only had chart review data for UM cases, and thus, the validity of exclusion criteria testing was only conducted in a single EHR.

There are several strengths of the eCQMs. First, both eCQMs were tested and validated in 2 EHRs representing 111 hospitals. Second, we assessed validity using multiple methods including comparing eCQM data to chart review. Finally, use of chart review versions of these eCQMs in HMS resulted in improvements in duration and empiric selection of antibiotics as well as outcomes for patients with CAP. Having eCQMs such as these be part of CMS’ Promoting Interoperability Program might further motivate EHR vendors to help implement the eCQMs, improving standardization and feasibility. This could allow antibiotic stewardship programs to use the data to inform their efforts and lead to further uses of the measure at jurisdictional or national levels.

## CONCLUSIONS

We developed highly important, reliable, valid, and feasible eCQMs of antibiotic use for hospitalized adults with uncomplicated CAP. These measures, both endorsed by PQM and on CMS’ Measures Under Consideration list, may be used in hospitals seeking to improve antibiotic use for CAP. Prospective studies in other environments that are evaluating the effect of interventions based on these metrics will be key to determining whether these eCQMs can positively affect outcomes generally.

## Supplementary Material

ciag273_Supplementary_Data

## Supplementary Data

Supplementary materials are available at *Clinical Infectious Diseases* online. Consisting of data provided by the authors to benefit the reader, the posted materials are not copyedited and are the sole responsibility of the authors, so questions or comments should be addressed to the corresponding author.
