# Supplementary material for: Electronic Clinical Quality Measures of Antibiotic Use in Hospitalized Adults With Uncomplicated Community-Acquired Pneumonia
Source: Clin Infect Dis. 2026 Apr 21;83(2):e251–9. doi: 10.1093/cid/ciag273 (PMC13537145; doi:10.1093/cid/ciag273)
Supplement: Supplementary file 1 [file ciag273_supplementary_data.docx]

**Supplemental Appendix**

**Electronic Clinical Quality Measures of Antibiotic Use in Hospitalized Adults with Uncomplicated Community-acquired Pneumonia**

| **Table of Contents** | **Page** |
| --- | --- |
| **Measure Specifications for Excess Antibiotic Duration for Adult Hospitalized Patients with Uncomplicated CAP eCQM** | **2** |
| **Measure Specifications for Inappropriately Broad Empiric Antibiotic Selection for Adult Hospitalized Patients with Uncomplicated CAP eCQM** | **4** |
| **eTable 1**. Members of Technical Expert Panels (TEP) and the organizations they represented for duration and empiric measures | **6** |
| **eTable 2.** Scores for duration and empiric therapy eCQMs across 3 cohorts | **6** |
| **eTable 3.** Accountable entity-level reliability testing results and performance scores by decile across 109 VA hospitals – Duration eCQM | **7** |
| **eTable 4.** Accountable entity-level reliability testing results and performance scores by decile across 109 VA hospitals – Empiric eCQM | **7** |
| **eTable 5.** Minimum annual number of qualifying cases needed for the denominator to reach each target reliability level at a given facility | **7** |
| **eTable 6**. Total antibiotic duration comparison for UM HMS chart review data (reference standard) to UM eCQM in 450 CAP patients | **8** |
| **eTable 7.** Inpatient antibiotic duration comparison for UM HMS chart review data (reference standard) to UM eCQM in 450 CAP patients | **8** |
| **eTable 8.** Discharge antibiotic duration comparison for UM HMS chart review data (reference standard) to UM eCQM in 450 CAP patients | **9** |
| **eTable 9.** Comparison of empiric antibiotic assessment for UM HMS chart review data (reference standard) to UM eCQM in 540 CAP patients | **9** |
| **eTable 10**. Comparison of denominator exclusions to define hospitalized non-ICU adults with CAP | **10** |
| **eTable 11.** Comparison of denominator exclusions to narrow population to uncomplicated CAP | **10** |
| **eTable 12.** Comparison of denominator exclusions specific to calculation of duration | **10** |
| **eTable 13.** Additional denominator exclusions specific to empiric measure | **11** |
| **eFigure 1.** Correlation between antibiotic and broad empiric antibiotic measures | **11** |
| **eTable 14**. Data elements with feasibility challenges identified during measure development and resulting decisions for use in both eCQMs | **12** |
| **Technical Expert Panel (TEP) survey results – Duration Measure** | **14** |
| **Technical Expert Panel (TEP) survey results – Empiric Measure** | **15** |
| **eTable 15.** Respiratory antibiotics for CAP diagnosis | **17** |
| **References** | **19** |

**Measure Specifications for Excess Antibiotic Duration for Adult Hospitalized Patients with Uncomplicated CAP**

The Excess Antibiotic Duration for Adult Hospitalized Patients with Uncomplicated Community-Acquired Pneumonia measure is a process measure representing the annual percentage of hospitalized adults with uncomplicated community-acquired pneumonia who receive unsupported excess antibiotic duration. The measure will be calculated using electronic health record (EHR) data and is intended for use at the facility level. Including the measures in the CMS Promoting Interoperability Program could improve their adoption, potentially enabling CAP-directed stewardship efforts nationally.

**Numerator:**

From the denominator population, identify patients that qualify for a 5-day duration according to national guidelines who received excess antibiotic duration (defined as >/= 7 days of total antibiotic therapy including inpatient and discharge antibiotics).

**Denominator:**

Identify all adult patients with an inpatient (or observation) non-intensive care unit (ICU) hospitalization in which the discharge diagnosis includes pneumonia or sepsis AND respiratory failure who received a respiratory antibiotic (eTable 15, below) within 48 hours of hospitalization, received chest imaging within +/- 3 days of the hospital encounter, were not transferred from another hospital, and do not have a concurrent infection. Exclude patients with complicated pneumonia and indications for a longer duration.

**Inclusion Criteria:**

- ICD10 discharge code for pneumonia or sepsis AND respiratory failure
- Received a respiratory antibiotic within the first 48 hours of hospitalization
- Age > 18 years on admission
- Admitted as inpatient or in observation awaiting inpatient admission
- Did not die in first 48 hours of hospitalization
- No time spent in an ICU in first 48 hours of hospitalization
- Not admitted from another acute care hospital or long-term acute care facility
- No concurrent infection during hospitalization by ICD 10
- Chest imaging within 3 days of admission

**Denominator Exclusions:**

**To identify uncomplicated CAP patients: Exclude if:**

- On mechanical ventilation in first 48 hours
- Absolute neutrophil count <500 cells/uL
- Cystic fibrosis
- Bronchiectasis
- Human immunodeficiency virus (HIV)
- Tracheostomy
- Transplant code in prior year (to identify lifetime transplant)
- Hematologic malignancy
- Pulmonary complication (empyema, lung abscess, necrotizing pneumonia)

**Exclude cases in which duration is unknown:**

- Died during hospitalization
- Discharged to another hospital
- Discharged to inpatient/home hospice
- Antibiotic prescribed at discharge, but duration is missing from record

**Exclude conditions requiring longer duration:**

- Transferred to ICU during hospitalization
- Bacteremic with non-skin commensal
- *Staphylococcus aureus* in respiratory culture
- *Pseudomonas aeruginosa* in respiratory culture
- Legionella pneumonia
- Time to clinical stability (afebrile and normotensive or discharged) > 5 days

**Exclude conditions where treatment likely not for uncomplicated CAP:**

- <3 days of antibiotics
- >14-day total antibiotic duration

Note–all times are calculated from arrival to the emergency department

**Measure Specifications for Inappropriately Broad Empiric Antibiotic Selection for Adult Hospitalized Patients with Uncomplicated CAP eCQM**

The inappropriately broad empiric antibiotic selection eCQM is a process measure representing the annual percentage of hospitalized adults with uncomplicated community-acquired pneumonia. Here, we defined “inappropriately broad” as any antibiotic therapy targeting methicillin-resistant *Staphylococcus aureus* (MRSA) or *P. aeruginosa* in patients without risk factors for one of those organisms. The measure will be calculated using EHR data and is intended for use at the facility level. Including the measures in the CMS Promoting Interoperability Program could improve their adoption, potentially enabling CAP-directed stewardship efforts nationally.

**Numerator:**

From the denominator population, identify patients without risk factors for methicillin-resistant *Staphylococcus aureus* (MRSA) or *P. aeruginosa* who received empiric antibiotics (within the first 48 hours of emergency department arrival) targeting MRSA or *P. aeruginosa.*

**Denominator:**

Identify all adult patients with an inpatient (or observation) non-ICU hospitalization in which the discharge diagnosis includes pneumonia or sepsis/respiratory failure who received a respiratory antibiotic (eTable 15, below) within 48 hours of hospitalization, received chest imaging within +/- 3 days of the hospital encounter, were not transferred from another hospital, and do not have a concurrent infection. Restrict to patients with uncomplicated pneumonia and without risk factors for MRSA or *Pseudomonas aeruginosa*  (i.e., positive respiratory culture for MRSA/pseudomonas in prior year or hospitalization within the last 90 days with receipt of IV antibiotics).^29,30^

**Inclusion Criteria:**

- ICD10 discharge code for pneumonia or sepsis AND respiratory failure
- Received a respiratory antibiotic within the first 48 hours of hospitalization
- Age > 18 years on admission
- Admitted as inpatient or in observation awaiting inpatient admission
- Did not die in first 48 hours of hospitalization
- No time spent in an ICU in first 48 hours of hospitalization
- Not admitted from another acute care hospital or long-term acute care facility
- No concurrent infection during hospitalization by ICD 10
- Chest imaging within 3 days of admission

*note–all times are calculated from arrival to the emergency department

**Denominator Exclusions:**

**To identify uncomplicated CAP patients: Exclude if:**

- On mechanical ventilation in first 48 hours
- Absolute neutrophil count <500 cells/uL
- Cystic fibrosis
- Bronchiectasis
- Human immunodeficiency virus (HIV)
- Tracheostomy
- Transplant in prior year
- Hematologic malignancy
- Pulmonary complication (empyema, lung abscess, necrotizing pneumonia)

**Exclude patients with risk factors for MRSA/*Pseudomonas aeruginosa*:**

- *P. aeruginosa* in respiratory culture in prior year
- MRSA in respiratory culture in prior year

**Exclude severe pneumonia AND prior hospitalization with IV antibiotics in past 3 months**

**Severe pneumonia** defined by ≥2 of the following on a single day in the first 48 hours of encounter:

- Respiratory rate >30 breaths/min
- ≥5L oxygen or SpO2<90
- White blood cell (WBC) <4000 cell/uL
- Blood urea nitrogen (BUN) >20 mg/dL
- Platelets <100 K/uL
- Temperature <36 C
- Systolic blood pressure (SBP) <80 mmHg

AND

**Prior hospitalization with IV antibiotics in past 3 months**

Note–all times are calculated from arrival to the emergency department

**eTable 1.** Members of Technical Expert Panels (TEP) and the organizations they represented for duration and empiric measures

| Name | Organization | Duration | Empiric |
| --- | --- | --- | --- |
| Julio Ramirez, MD | Infectious Disease Society of America (IDSA) | x | x |
| Dan Morgan, MD, MS | Society of Healthcare of America (SHEA) | x |  |
| Michelle Dardis, RN | The Joint Commission | x | x |
| Michael Pulia, MD, MS | Emergency Medicine; University of Wisconsin Madison | x | x |
| Marc Meyer, PharmD | American Society of Health-System Pharmacists (ASHP) | x | x |
| Jason Pogue, PharmD, BCPS, BCIDP | Society of Infectious Diseases Pharmacists (SIDP) | x | x |
| Kristina Crothers, MD | American Thoracic Society (ATS) | x | x |
| Mark Metersky, MD, FCCP, FACP | American Thoracic Society (ATS) | x |  |
| Liam Sullivan, DO | Society of Healthcare of America (SHEA) |  | x |
| Peter Lindenauer, MD, MSc, MHM, FACP | Society of Hospital Medicine (SHM) |  | x |
| Laura Feemster, MD, MS | American Thoracic Society (ATS) |  | x |

**eTable 2.** Scores for duration and empiric therapy eCQMs across 3 cohorts

| ***** | **UM**  9/29/2015-12/22/2021  (n=3,417) | **UU**  1/1/2021-5/30/2022  (n=313) | **VA**  1/1/2022-6/30/2024  (n=28,238) |
| --- | --- | --- | --- |
| Median (IQR) Total Days of Antibiotic Use | 7 (5-8.5) | 6 (4-7) | 6 (5-7) |
| Median (IQR) Inpatient Duration | 4 (3-5) | 3 (3-5) | 4 (3-5) |
| **Duration eCQM score** (received ≥7 days total duration) | 1,861 (54.5%) | 125 (39.9%) | 11,232 (39.8%) |
| ***** | **UM**  (n=5,553) | **UU**  (n=619) | **VA**  (n=47,034) |
| **Empiric** **eCQM** **score** (received EITHER anti-MRSA or anti-pseudomonal therapy in first 48 hours**)** | 3,105 (55.9%) | 103 (16.6%) | 14,407 (30.6%) |
| Received anti-MRSA therapy in first 48 hours | 2,490 (44.8%) | 66 (10.7%) | 7,434 (15.8%) |
| Received anti-pseudomonal therapy in first 48 hours | 2,933 (52.8%) | 65 (10.5%) | 13,165 (28.0%) |

*cell intentionally left empty

Abbreviations: eCQM, electronic clinical quality measure; IQR, interquartile range; UM, University of Michigan; UU, University of Utah; VA, Veterans Affairs; IQR, interquartile range; MRSA, methicillin-resistant *Staphylococcus aureus*

**eTable 3.** Accountable entity-level reliability testing results and performance scores by decile across 109 VA healthcare systems – Duration eCQM

| ***** | **Overall** | **Min** | **Decile**  **1** | **Decile**  **2** | **Decile**  **3** | **Decile**  **4** | **Decile**  **5** | **Decile**  **6** | **Decile**  **7** | **Decile**  **8** | **Decile**  **9** | **Decile**  **10** | **Max** |
| --- | --- | --- | --- | --- | --- | --- | --- | --- | --- | --- | --- | --- | --- |
| Reliability | 99.9% | 30.2% | 62.7% | 84.3% | 90.1% | 91.6% | 92.7% | 93.7% | 94.4% | 95.5% | 96.4% | 97.3% | 98.3% |
| Mean Performance Score | 39.8% | 20.0% | 45.5% | 46.2% | 34.2% | 42.7% | 33.4% | 36.2% | 38.1% | 37.3% | 39.7% | 44.0% | 41.4% |
| N of Entities | 109 | 1 | 11 | 11 | 11 | 11 | 10 | 11 | 11 | 11 | 11 | 11 | 1 |
| N of Persons / Encounters | 28238 | 5 | 348 | 1026 | 1485 | 1851 | 1953 | 2635 | 3075 | 3845 | 4784 | 7236 | 988 |

*cell intentionally left empty

**eTable 4.** Accountable entity-level reliability testing results and performance scores by decile across 109 VA healthcare systems – Empiric eCQM

| ***** | **Overall** | **Min** | **Decile**  **1** | **Decile**  **2** | **Decile**  **3** | **Decile**  **4** | **Decile**  **5** | **Decile**  **6** | **Decile**  **7** | **Decile**  **8** | **Decile**  **9** | **Decile**  **10** | **Max** |
| --- | --- | --- | --- | --- | --- | --- | --- | --- | --- | --- | --- | --- | --- |
| Reliability | 99.9% | 29.1% | 62.5% | 81.7% | 86.6% | 88.3% | 90.8% | 91.5% | 92.5% | 94.2% | 95.1% | 96.3% | 97.7% |
| Mean Performance Score | 30.6% | 12.5% | 19.2% | 28.7% | 30.4% | 31.7% | 26.6% | 30.7% | 32.7% | 28.5% | 32.1% | 31.9% | 30.6% |
| N of Entities | 109 | 1 | 11 | 11 | 11 | 11 | 10 | 11 | 11 | 11 | 11 | 11 | 1 |
| N of Persons / Encounters | 47034 | 8 | 591 | 1777 | 2610 | 3127 | 3315 | 4298 | 5044 | 6353 | 8161 | 11758 | 1564 |

*cell intentionally left empty

**eTable 5.** Minimum annual number of qualifying cases needed for the denominator to reach each target reliability level at a given facility

| **Reliability** | **Duration Measure (n)** | **Empiric Measure (n)** |
| --- | --- | --- |
| 0.6 (acceptable) | 13 | 36 |
| 0.7 (acceptable/good) | 19 | 56 |
| 0.8 (good/excellent) | 49 | 96 |
| 0.9 (excellent) | 110 | 216 |

Calculations were derived from clinical data from 109 Veterans Affairs (VA) healthcare systems between Jan 2022 – June 2024 (n=28,238 and n=47,034 hospitalized pneumonia patients for duration and empiric measures, respectively). Signal-to-noise analysis performed using a mixed-effect logistic model enabled calculation of the hospital variance (signal), total variance, and within hospital variance (noise). An intraclass correlation was calculated based on the hospital variance and the within hospital variance using the Spearman Brown formula allowed calculation of measure reliability for the entire hospital cohort and quantification of the minimum number of cases to achieve reliability thresholds.

**eTable 6.** Total antibiotic duration comparison for UM HMS chart review data (reference standard) to UM eCQM in 450 CAP patients

| Total Duration | UM HMS  <5 days, n=15 | UM HMS  5 days, n=67 | UM HMS  6 days, n=90 | UM HMS  7 days, n=85 | UM HMS  8 days, n=76 | UM HMS  >8 days, n=117 |
| --- | --- | --- | --- | --- | --- | --- |
| UM eCQM  <5 days, n=17 | 14 (3.1%) | 0 | 0 | 1 (0.2%) | 0 | 2 (0.4% |
| UM eCQM  5-5.5 days, n=70 | 0 | 62 (13.8%) | 5 (1.1%) | 3 (0.7%) | 0 | 0 |
| UM eCQM  6-6.5 days, n=89 | 0 | 4 (0.9%) | 81 (18.0%) | 1 (0.2%) | 0 | 3 (0.7%) |
| UM eCQM  7-7.5 days, n=88 | 1 (0.2%) | 0 | 3 (0.7%) | 78 (17.3%) | 3 (0.7%) | 3 (0.7%) |
| UM eCQM  8-8.5 days, n=78 | 0 | 1 (0.2%) | 1 (0.2%) | 1 (0.2%) | 71 (15.8%) | 4 (0.9%) |
| UM eCQM  >8.5 days, n=108 | 0 | 0 | 0 | 1 (0.2%) | 2 (0.4%) | 105 (23.3%) |

411/450 (91.3%) in green were accurate within a ½ day; 434/450 (96.4%) green/yellow were accurate within 1.5 days

Abbreviations: eCQM, electronic clinical quality measure; CAP, community-acquired pneumonia; UM, University of Michigan; HMS, Michigan Hospital Medicine Safety Consortium

**eTable 7.** Inpatient antibiotic duration comparison for UM HMS chart review data (gold standard) to UM eCQM in 450 CAP patients

| Inpatient Duration | UM HMS  <5 days | UM HMS  5 days | UM HMS  6 days | UM HMS  7 days | UM HMS  8 days | UM HMS  >8 days |
| --- | --- | --- | --- | --- | --- | --- |
| UM eCQM  <5 days | 278 (61.2%) | 1 (0.2%) | 3 (0.7%) | 0 | 0 | 0 |
| UM eCQM  5-5.5 days | 0 | 82 (18.1%) | 1 (0.2%) | 1 (0.2%) | 0 | 1 (0.2%) |
| UM eCQM  6-6.5 days | 0 | 1 (0.2%) | 47 (10.4%) | 2 (0.4%) | 0 | 0 |
| UM eCQM  7-7.5 days | 0 | 0 | 2 (0.4%) | 18 (4.0%) | 1 (0.2%) | 2 (0.4%) |
| UM eCQM  8-8.5 days | 0 | 0 | 0 | 0 | 11 (2.4%) | 0 |
| UM eCQM  >8.5 days | 0 | 0 | 0 | 0 | 0 | 3 (0.7%) |

439/454 (96.7%) in green were accurate within a ½ day; 447/454 (98.5%) green/yellow were accurate within 1.5 days

Abbreviations: eCQM, electronic clinical quality measure; CAP, community-acquired pneumonia; UM, University of Michigan; HMS, Michigan Hospital Medicine Safety Consortium

**eTable 8.** Discharge antibiotic duration comparison for UM HMS chart review data (reference standard) to UM eCQM in 450 CAP patients

| Discharge Duration | UM HMS  0 days | UM HMS  1 day | UM HMS  2 days | UM HMS  3 days | UM HMS  4 days | UM HMS  5 days | UM HMS  6 days | UM HMS  >6 days |
| --- | --- | --- | --- | --- | --- | --- | --- | --- |
| UM eCQM  0-0.5 days | 85 (18.9%) | 0 | 0 | 1 (0.2%) | 1 (0.2%) | 0 | 2 (0.4%) | 0 |
| UM eCQM  1-1.5 | 1 (0.2%) | 28 (6.1%) | 2 (0.4%) | 2 (0.4%) | 0 | 0 | 0 | 0 |
| UM eCQM  2-2.5 | 0 | 0 | 52 (11.4%) | 1 (0.2%) | 1 (0.2%) | 2 (0.4%) | 0 | 0 |
| UM eCQM  3-3.5 | 1 (0.2%) | 1 (0.2%) | 2 (0.4%) | 70 (15.3%) | 0 | 0 | 0 | 1 (0.2%) |
| UM eCQM  4-4.5 | 1 (0.2%) | 1 (0.2%) | 1 (0.2%) | 1 (0.2%) | 67 (14.6%) | 0 | 0 | 0 |
| UM eCQM  5-5.5 | 1 (0.2%) | 0 | 1 (0.2%) | 0 | 1 (0.2%) | 74 (16.2%) | 0 | 0 |
| UM eCQM  6-6.5 | 0 | 0 | 1 (0.2%) | 0 | 0 | 0 | 19 (4.2%) | 0 |
| UM eCQM  >6.5 | 3 (0.7%) | 0 | 0 | 1 (0.2) | 0 | 1 (0.2%) | 0 | 32 (7.0%) |

427/458 (93.2%) in green were accurate within a ½ day; 435/458 (95.0%) green/yellow were accurate within 1.5 days

Abbreviations: eCQM, electronic clinical quality measure; CAP, community-acquired pneumonia; UM, University of Michigan; HMS, Michigan Hospital Medicine Safety Consortium

**eTable 9.** Comparison of empiric antibiotic assessment for UM HMS chart review data (reference standard) to UM eCQM in 540 CAP patients

| * | UM HMS  Neither anti-MRSA/ pseudomonal (n=234) | UM HMS  Only anti-MRSA (n=8) | UM HMS  Only anti-pseudomonal (n=54) | UM HMS  Both anti-MRSA/ pseudomonal (n=244) |
| --- | --- | --- | --- | --- |
| UM eCQM  Neither | 233 (99.6%) | 3 (37.5%) | 8 (14.8%) | 2 (0.8%) |
| UM eCQM  Only anti-MRSA | 1 (0.4%) | 5 (62.5%) | 0 | 0 |
| UM eCQM  Only anti-pseudomonal | 0 | 0 | 46 (85.2%) | 4 (1.6%) |
| UM eCQM  Both | 0 | 0 | 0 | 238 (97.5%) |

Abbreviations: eCQM, electronic clinical quality measure; CAP, community-acquired pneumonia; UM, University of Michigan; HMS, Michigan Hospital Medicine Safety Consortium; MRSA, methicillin-resistant *Staphylococcus aureus*

**eTable 10.** Comparison of denominator exclusions to define hospitalized non-ICU adults with CAP

| **Exclusion** | **Michigan**  **(n=49,205)** | **Utah**  **(n=5,909)** | **VA**  **(n=102,416)** |
| --- | --- | --- | --- |
| Under 18 on admission | 8,905 (18.1%) | 0 | 0 |
| Patient class NOT inpatient or observation | 4,289 (8.7%) | 908 (15.4%) | NA |
| Died in the first 48 hours of hospitalization | 709 (1.4%) | 133 (2.3%) | 418 (0.4%) |
| Any time in ICU in the first 48 hours of hospitalization | 10,910 (22.2%) | 2391 (40.5%) | 17512 (17.1%) |
| Admitted from another hospital or long-term acute care facility | 1,272 (2.6%) | 1,506 (25.5%) | 7153 (7.0%) |
| Concurrent infection by ICD10 | 7,903 (16.1%) | 2,424 (41.0%) | 24719 (24.1%) |
| Chest imaging required (day -3 to day 3 of admission) | 465 (0.9%) | 726 (12.3%) | 2189 (2.1%) |
| **Ending population** | **10,366 (21.1%)** | **1049 (17.8%)** | **60,943 (59.5%)** |

Abbreviations: ICU, intensive care unit; CAP, community-acquired pneumonia

**eTable 11.** Comparison of denominator exclusions to narrow population to uncomplicated CAP

| **Exclusion** | **Michigan (n=10,366)** | **Utah**  **(n=1049)** | **VA**  **(n=60,943)** |
| --- | --- | --- | --- |
| Cystic Fibrosis | 286 (2.8%) | 19 (1.8%) | 18 (0.0%) |
| On mechanical ventilation in first 48 hours of hospitalization | 690 (6.7%) | 46 (4.4%) | NA |
| Absolute neutrophil count <500 cells/uL | 443 (4.3%) | 81 (7.7%) | 111 (0.2%) |
| Bronchiectasis | 791 (7.6%) | 58 (5.5%) | 2803 (4.6%) |
| HIV (irrespective of CD4) | 52 (0.5%) | 26 (2.5%) | 978 (1.6%) |
| Tracheostomy | 384 (3.7%) | 20 (1.9%) | 633 (1.0%) |
| Transplant in prior year | 1522 (14.7%) | 150 (14.3%) | 1478 (2.4%) |
| Hematologic malignancy | 1461 (14.1%) | 180 (17.2%) | 4762 (7.8%) |
| Pulmonary complication: | 263 (2.5%) | 38 (3.6%) | 1698 (2.8%) |
| **Ending population** | **6,285 (60.6%)** | **655 (62.4%)** | **49,774 (81.7%)** |

Abbreviations: HIV, Human Immunodeficiency Virus; CD4, CD4 T lymphocytes

**eTable 12.** Comparison of denominator exclusions specific to calculation of duration

| **Exclusion** | **Michigan (n=6285)** | **Utah**  **(n=655)** | **VA**  **(n 49774)** |
| --- | --- | --- | --- |
| Cannot calculate duration | * | * | * |
| Died during hospitalization | 223 (3.5%) | 7 (1.1%) | 830 (1.7%) |
| Discharged to another hospital | 20 (0.3%) | 6 (0.9%) | 2994 (6.0%) |
| Discharged to inpatient/home hospice | 284 (4.5%) | 30 (4.6%) | 333 (0.7%) |
| Appropriate Duration May be Longer | * | * | * |
| Transferred to ICU during hospitalization | 253 (4.0%) | 44 (6.7%) | 4262 (8.6%) |
| Bacteremic with non-skin commensal | 317 (5.0%) | 0 | 1085 (2.8%) |
| *Staphylococcus aureus* grew from respiratory culture during hospitalization | 123 (2.0%) | 3 (0.5%) | 810 (1.6%) |
| *Pseudomonas aeruginosa* grew from respiratory culture during hospitalization | 92 (1.5%) | 2 (0.3%) | 665 (1.4%) |
| *Legionella* pneumonia | 24 (0.4%) | 4 (0.6%) | 583 (1.2%) |
| Time to clinical stability >5 days | 779 (12.4%) | 2 (0.3%) | 209 (1.3%) |
| Condition likely not CAP | * | * | * |
| Received <3 days of antibiotics | 1166 (18.6%) | 192 (29.3%) | 13061 (25.4%) |
| >14-day total antibiotic duration | 414 (6.6%) | 92 (14.0%) | 1561 (3.2%) |
| **Ending population** | **3437 (54.7%)** | **313 (47.8%)** | **28,238 (56.7%)** |

*cell intentionally left empty

Abbreviations: ICU, intensive care unit; CAP community-acquired pneumonia

**eTable 13.** Additional denominator exclusions specific to empiric measure

| **Exclusion** | **Michigan (n=6,285)** | **Utah**  **(n=655)** | **VA**  **(n=49,774)** |
| --- | --- | --- | --- |
| **Risk factors for MRSA/*Pseudomonas aeruginosa* from prior cultures; culture data from year prior** | | | |
| Positive respiratory culture for *Pseudomonas* *aeruginosa* in prior year | 68 (1.1%) | 0 | 695 (1.4%) |
| Positive respiratory culture for methicillin-resistant *Staphylococcus aureus* in prior year | 39 (0.6%) | 0 | 294 (0.6%) |
| **Patients with severe pneumonia (see definition below)** | | | |
| Severe pneumonia + prior hospitalization with intravenous antibiotic in prior 3 months | 677 (10.8%) | 36 (5.5%) | 2233 (4.5%) |
| **Ending population** | **5,553 (88.4%)** | **619 (94.5%)** | **48742 (97.9%)** |

**eFigure 1.** Correlation between antibiotic and broad empiric antibiotic measures

MDRO= Multi-Drug-Resistant Organisms

Patient data from 109 VA healthcare systems between January 1, 2022 and June 30, 2024. Each hospital is represented as a dot with larger hospitals having larger dots.

**eTable 14.** Data elements with feasibility challenges identified during measure development and resulting decisions for use in both eCQMs

| **Data Element** | **Planned Use** | **Feasibility Challenge** | **Decision and Rationale** |
| --- | --- | --- | --- |
| **Duration eCQM** | | | |
| Mon/Wed/Fri antibiotics | To exclude chronic suppressive antibiotics | The VA VistA EHR did not capture this type of dosing in a structured field; would present an undue burden to capture the data | Dropped. Testing in UM and UU systems found that 0.5% of patients had this type of dosing and 59% of those patients were excluded for another reason |
| Time to clinical stability >5 days | To exclude patients requiring longer antibiotic durations | National guidelines and clinical trials^11,25^ define clinical stability as being afebrile (≤37.8 °C) AND ≤1 of the following: heart rate ≥100 beats per minute; respiratory rate ≥24 breaths per minute; hypoxic (SpO2 <90% or PaO2 <60 mmHg), and SBP<90 mmHg. TEP expressed concerns about feasibility for assessing this definition, particularly for hospitals with fewer information technology resources (e.g., critical access hospitals) | We adopted a simpler and feasible definition of clinical stability: afebrile (max temperature for the calendar day ≤37.8 °C) and normotensive (lowest systolic blood pressure for the calendar day ≥90 mmHg) OR patient was discharged. When tested in the UM dataset, the modified clinical stability definition had sensitivity of 29% and specificity of 98%. We also assessed the impact of low sensitivity: using the original criteria, 53.7% (1734/3227) of patients were classified as having excess duration vs. 54.5% (1861/3417) using the modified definition. This difference did not change UM decile of performance for the measure. |
| **Empiric eCQM** | | | |
| Criteria for severe pneumonia | To exclude patients with severe/ complicated pneumonia | The 2019 ATS/IDSA Guidelines^24,25^ define severe pneumonia as having either one major criterion or three or more minor criteria; several data elements are not captured in structured fields | Redefined as ≥ 2 of the following elements from structured data fields: respiratory rate ≥30 breaths/minute; ≥5L oxygen or SpO2<90; BUN ≥ 20 mg/dL; WBC <4000 cells/uL; Platelets <100,000/uL; Temperature <36 C; SBP <80 mmHg. When tested in the UM dataset, the modified severity definition had sensitivity of 87% and specificity of 78% |
| **Both eCQMs** | | | |
| CD4 count (+ diagnosis of HIV) | Exclude patients with acquired immunodeficiency | CD4 results located in multiple different lab tests, often in unstructured fields; no ICD10 codes to distinguish acquired immunodeficiency syndrome from HIV | Redefined as excluding all patients with HIV (regardless of CD4 count), because 1) patients with HIV were uncommon in all three systems prior to other exclusions (0.5% UM, 1.6% VA; 2.1% UU), and 2) HIV can be identified by ICD10 diagnostic codes alone |
| Intravenous antibiotics at discharge | Exclude patients with severe/ complicated pneumonia | VA Vista EHR did not capture IV antibiotics after discharge in a structured field; presents an undue burden to capture the data | Dropped. UM data showed that only 2.2% of patients had IV antibiotics at discharge, of which 71% were excluded for an alternative reason; therefore, this exclusion has minimal impact on measure performance |

Abbreviations: Mon/Wed/Fri, Monday/Wednesday/Friday; VA VistA, Veterans Affairs Veterans Health Information Systems and Technology; EHR, electronic health record; UM, University of Michigan; UU, University of Utah; SpO2, peripheral oxygen saturation; PaO2, partial pressure of oxygen; SBP, systolic blood pressure; TEP, technical expert panel; ATS/IDSA, American Thoracic Society/Infectious Diseases Society of America; BUN, blood urea nitrogen; WBC, white blood cell; IV, intravenous; MRSA, methicillin-resistant *Staphylococcus aureus*; CD4, CD4 T lymphocytes; HIV, Human Immunodeficiency Virus

**Technical Expert Panel (TEP) survey results – Duration Measure**

| Question | | | | Response | N (%) |
| --- | --- | --- | --- | --- | --- |
| *How important do you think it is to develop an electronic measure of the duration of antibiotic therapy for persons diagnosed with community-acquired pneumonia (CAP)?* | | | | *Very Important*  *Important*  *Neutral*  *Unimportant*  *Very Unimportant* | 6 (75%)  2 (25%)  0  0  0 |
| Comments: | “Excessive treatment is very common and this could reduce antibiotic burden and improve patient safety without worse outcomes”  “The measure would support a standard, operational definition and practice for antibiotic treatment and prescribing.”  “This is one of the easiest, most evidence-based interventions that can be made. one that clearly is safe to do and there is clear harm if we do not optimize.”  “There is a large gap in adherence to recommended length of therapy”  “on a population level, can decrease antibiotic use significantly” | | | | |
| *The measure as specified can be used to distinguish between better and worse performing hospitals.* | | | | *Very Likely*  *Likely*  *Neutral*  *Unlikely*  *Very Unlikely* | 2 (25%)  6 (75%)  0  0  0 |
| Comments: | “This is a great attempt to accomplish decrease DOT for pneumonia. Compressing to make it extractable and provide good data is difficult. Certainly HMS has been able to show it works.”  “Computationally, calculating clinical stability could be resource intensive for EHR vendors/data intermediaries, due to the large volume of vitals data and complex logic to connect values. Suggest seeking vendor feedback as part of the feasibility evaluation for this data element.” | | | | |
| *Consensus from today's TEP suggests that the modified measure should be: For all patients who meet CAP inclusion criteria, >6 days is considered inappropriate/excess therapy (language to be discussed below). Patients who did not achieve clinical stability by day 5 would be excluded. Do you agree with the proposed criteria for the appropriate duration of therapy for pneumonia?* | | | | *Strongly Agree*  *Agree*  *Neutral*  *Disagree*  *Strongly Disagree* | 5 (63%)  3 (37%)  0  0  0 |
| Comments: | | “That seem[s] to simplify the complexity and still produce the end result” | | | |
| *Are there any key data elements you believe are missed or not appropriately used in the determination of the appropriate duration of therapy for CAP measure?*” | | | |  |  |
| Comments: | | | “The clinical stability definition is computationally and operationally complex and I am concerned that the initial pilot results will not play out in a larger population with disparate EHRs- particularly due to outlier vital signs values (elevated HR on ambulation) and data completeness/timeliness of supplemental oxygen documentation, particularly when oxygen orders are PRN.”  “It may be difficult for some facilities to pull data to determine stability.” | | |

| *What unintended consequences do you see/foresee to using the proposed measure of the duration of therapy for CAP to guide care improvement, if any?* | |  |  |
| --- | --- | --- | --- |
| Comments: | “Given how few patients do not stabilize by day 5, and the fact that they will be clearly excluded makes me comfortable that we won’t be fostering a thought process where undertreatment is preferred.”  “Hard to see that there would be” | | |

**Technical Expert Panel (TEP) survey results – Empiric Measure**

| Question | | | | Response | N (%) |
| --- | --- | --- | --- | --- | --- |
| *How important do you think it is to develop an electronic measure of the quality of empiric antibiotic therapy for persons diagnosed with pneumonia?* | | | | *Very Important*  *Important*  *Neutral*  *Unimportant*  *Very Unimportant* | 3 (37%)  5 (63%)  0  0  0 |
| *How do you think the measure should be simplified?* | | | |  |  |
| Comments: | “Eliminating anyone who qualifies for MDRO therapy & focusing on pts who do not have risk factors for MDRO therapy”  “As proposed above- exclude those with indication for MDR therapy and focus on overuse” | | | | |
| *One suggestion from the 4/26 TEP was to collapse the tiers to assess ONLY patients for whom MDRO therapy is not recommended (i.e., exclude those with risk factors for MDRO - see table for details). Do you agree with this new assignment of tiers?* | | | | *Strongly Agree*  *Agree*  *Neutral*  *Disagree*  *Strongly Disagree* | 5 (63%)  2 (25%)  1 (12%)  0  0 |
| Comments: | “…I think evaluating pts for whom MDRO therapy is not clearly indicated is cleaner and will make this metric easier and more fair. It certainly isn't perfect, but then again what quality metric is perfect!”  “I'm assuming you would set this up as a "lower is better" proportion measure, where only patients who received Tier 3, non-guideline concordant therapy, were included in the numerator. In this scenario, "Tier 1" would be in the denominator but not the numerator.” | | | | |
| *The simplified measure* can be used to distinguish between better and worse performing hospitals.* | | | | *Strongly Agree*  *Agree*  *Neutral*  *Disagree*  *Strongly Disagree* | 1 (14%)  4 (57%)  2 (28%)  0  0 |
| Comments: | | “There is no perfect measure here but I think simplifying the measure by taking out those who qualify for MDRO treatment would make it cleaner & more fair.”  “I think it's the best option - but there are trade offs needed for the measure to remain accurate while still being feasible to use.” | | | |
| *Are there any key data elements you believe are missed or not appropriately used in the determination as to whether therapy for MDRO is justified?* | | | | | |
| Comments: | | | “No- always concern of outside data not available at time of admission- but that can't be avoided.”  “I think pulling from the EHR is going to miss some people in whom MDRO therapy is justified because they have a history that is reported in text notes and is not retrievable in the EHR from prior medications and microbiology results. Diagnoses of bronchiectasis, severe COPD, other structural risk factors may also be missed if not coded during that hospitalization. We discussed the expectation that we don't think the measure should be 100%. | | |
| *What unintended consequences do you see/foresee to using the proposed measure of the selection of empiric therapy for CAP to guide care improvement?* | | | | | |
| Comments: | | | “By concentrating on overuse- you could swing to underuse - but suspect unlikely. The original tiered system just seems too complicated to actually be used in practice- even if valid.”  “Simplified: potential for undertreatment in patients suspected of MDRO who do not meet criteria. Both versions: Documentation burden to collect MRDO verification data elements” | | |

*At the time of the TEP, we presented the original, more complicated version of the measure that included 3 Tiers (“Guideline concordant” [recommended]; “Guideline discordant”; and “Potentially justified”) and included patients who qualified for MDRO therapy. Based on TEP discussions, we simplified the measure.

**eTable 15.** Respiratory antibiotics for CAP diagnosis

| **Code** | **Description** |
| --- | --- |
| 1009148 | ampicillin / sulbactam |
| 102745 | pivmecillinam hydrochloride |
| 1040005 | ceftaroline |
| 11124 | vancomycin |
| 1272 | aztreonam |
| 139462 | moxifloxacin |
| 1540825 | tedizolid |
| 1597614 | ceftolozane / tazobactam |
| 1603839 | avibactam / ceftazidime |
| 190376 | linezolid |
| 1927663 | delafloxacin |
| 1945217 | meropenem / vaborbactam |
| 19552 | cefprozil |
| 19711 | amoxicillin / clavulanate |
| 20481 | cefepime |
| 20489 | cefpodoxime |
| 2059269 | omadacycline |
| 2176 | cefaclor |
| 2177 | cefadroxil |
| 2180 | cefazolin |
| 2184 | cefoperazone |
| 2184141 | cilastatin / imipenem / relebactam |
| 2191 | ceftazidime |
| 2193 | ceftriaxone |
| 2194 | cefuroxime |
| 2198944 | lefamulin |
| 2231 | cephalexin |
| 2265702 | cefiderocol |
| 25033 | cefixime |
| 25037 | cefdinir |
| 2551 | ciprofloxacin |
| 2573160 | durlobactam / sulbactam |
| 2582 | clindamycin |
| 2625 | cloxacillin |
| 2675987 | cefepime / enmetazobactam |
| 2691334 | ceftobiprole medocaril sodium |
| 29561 | meropenem |
| 325642 | ertapenem |
| 34482 | cilastatin / imipenem |
| 384455 | tigecycline |
| 5690 | imipenem |
| 723 | amoxicillin |
| 733 | ampicillin |
| 74169 | piperacillin / tazobactam |
| 7623 | ofloxacin |
| 7980 | penicillin G |
| 7984 | penicillin V |
| 82122 | levofloxacin |

Note. vancomycin is included and coded separately. It is not included in the value set because only IV delivery is specific to CAP.

**References**

1.	Fridkin S, Baggs J, Fagan R, et al. Vital signs: improving antibiotic use among hospitalized patients. *MMWR Morbidity and mortality weekly report*. Mar 7 2014;63(9):194–200.

2.	Tamma PD, Avdic E, Li DX, Dzintars K, Cosgrove SE. Association of Adverse Events With Antibiotic Use in Hospitalized Patients. *JAMA Internal Medicine*. 2017;

3.	Madaras-Kelly KJ, Burk M, Caplinger C, et al. Total duration of antimicrobial therapy in veterans hospitalized with uncomplicated pneumonia: Results of a national medication utilization evaluation. *Journal of hospital medicine*. Dec 2016;11(12):832–839. doi:10.1002/jhm.2648

4.	Vaughn VM, Flanders SA, Snyder A, et al. Excess Antibiotic Treatment Duration and Adverse Events in Patients Hospitalized with Pneumonia: A Multihospital Cohort Study. *Annals of internal medicine*. Aug 6 2019;171(3):153–163. doi:10.7326/M18-3640

5.	Werner NL, Hecker MT, Sethi AK, Donskey CJ. Unnecessary use of fluoroquinolone antibiotics in hospitalized patients. *BMC infectious diseases*. 2011;11(1):187.

6.	Hecker MT, Aron DC, Patel NP, Lehmann MK, Donskey CJ. Unnecessary use of antimicrobials in hospitalized patients: current patterns of misuse with an emphasis on the antianaerobic spectrum of activity. *Archives of internal medicine*. Apr 28 2003;163(8):972–8. doi:10.1001/archinte.163.8.972

7.	Jernigan JA, Hatfield KM, Wolford H, et al. Multidrug-Resistant Bacterial Infections in U.S. Hospitalized Patients, 2012-2017. *N Engl J Med*. Apr 2 2020;382(14):1309–1319. doi:10.1056/NEJMoa1914433

8.	Bell BG, Schellevis F, Stobberingh E, Goossens H, Pringle M. A systematic review and meta-analysis of the effects of antibiotic consumption on antibiotic resistance. *BMC Infect Dis*. Jan 9 2014;14:13. doi:10.1186/1471-2334-14-13

9.	Gontjes KJ, Gibson KE, Lansing BJ, et al. Association of Exposure to High-risk Antibiotics in Acute Care Hospitals With Multidrug-Resistant Organism Burden in Nursing Homes. *JAMA Netw Open*. Feb 1 2022;5(2):e2144959. doi:10.1001/jamanetworkopen.2021.44959

10.	Global burden of bacterial antimicrobial resistance in 2019: a systematic analysis. *Lancet*. Feb 12 2022;399(10325):629–655. doi:10.1016/s0140-6736(21)02724-0

11.	Nelson RE, Hyun D, Jezek A, Samore MH. Mortality, Length of Stay, and Healthcare Costs Associated With Multidrug-Resistant Bacterial Infections Among Elderly Hospitalized Patients in the United States. *Clin Infect Dis*. Mar 23 2022;74(6):1070–1080. doi:10.1093/cid/ciab696

12.	Magill SS, Edwards JR, Beldavs ZG, et al. Prevalence of antimicrobial use in US acute care hospitals, May-September 2011. *JAMA*. Oct 8 2014;312(14):1438–46. doi:10.1001/jama.2014.12923

13.	Schrag SJ, Pena C, Fernandez J, et al. Effect of short-course, high-dose amoxicillin therapy on resistant pneumococcal carriage: a randomized trial. *JAMA*. Jul 4 2001;286(1):49–56. doi:10.1001/jama.286.1.49

14.	Wistrom J, Norrby SR, Myhre EB, et al. Frequency of antibiotic-associated diarrhoea in 2462 antibiotic-treated hospitalized patients: a prospective study. *The Journal of antimicrobial chemotherapy*. Jan 2001;47(1):43–50. doi:10.1093/jac/47.1.43

15.	Branch-Elliman W, O’Brien W, Strymish J, Itani K, Wyatt C, Gupta K. Association of Duration and Type of Surgical Prophylaxis With Antimicrobial-Associated Adverse Events. *JAMA Surgery*. 2019;154(7):590–598. doi:10.1001/jamasurg.2019.0569

16.	Jones BE, Ying J, Stevens V, et al. Empirical Anti-MRSA vs Standard Antibiotic Therapy and Risk of 30-Day Mortality in Patients Hospitalized for Pneumonia. *JAMA Intern Med*. Apr 1 2020;180(4):552–560. doi:10.1001/jamainternmed.2019.7495

17.	Attridge RT, Frei CR, Restrepo MI, et al. Guideline-concordant therapy and outcomes in healthcare-associated pneumonia. *Eur Respir J*. Oct 2011;38(4):878–87. doi:10.1183/09031936.00141110

18.	CDC. Core Elements of Hospital Antibiotic Stewardship Programs. US Department of Health and Human Services. CDC. Accessed August 3, 2022. https://www.cdc.gov/antibiotic-use/healthcare/pdfs/hospital-core-elements-H.pdf

19.	The Joint Commission. *R3 Report Issue 35: New and Revised Requirements for Antibiotic Stewardship* 2022. https://www.jointcommission.org/-/media/tjc/documents/standards/r3-reports/r3_antibioticstewardship_july2022_final.pdf

20.	QSO-22-20-Hospitals Infection Prevention and Control and Antibiotic Stewardship Program Interpretive Guidance Update (2022).

21.	NHSN. Antimicrobial Use and Resistance (AUR) Module. Centers for Disease Control. https://www.cdc.gov/nhsn/pdfs/pscmanual/11pscaurcurrent.pdf

22.	Partnership for Quality Measurement. Excess antibiotic duration for adult hospitalized patients with uncomplicated community-acquired pneumonia. Battelle. Accessed 5/1/2025, https://p4qm.org/measures/4540e

23.	Partnership for Quality Measurement. Inappropriately broad empiric antibiotic selecton for adult hospitalized patients with uncomplicated community-acquired pneumonia. Battelle. Accessed 5/1/25, https://p4qm.org/measures/4545e

24.	Mandell LA, Wunderink RG, Anzueto A, et al. Infectious Diseases Society of America/American Thoracic Society consensus guidelines on the management of community-acquired pneumonia in adults. *Clin Infect Dis*. Mar 1 2007;44 Suppl 2:S27–72. doi:10.1086/511159

25.	Metlay JP, Waterer GW, Long AC, et al. Diagnosis and Treatment of Adults with Community-acquired Pneumonia. An Official Clinical Practice Guideline of the American Thoracic Society and Infectious Diseases Society of America. *Am J Respir Crit Care Med*. Oct 1 2019;200(7):e45–e67. doi:10.1164/rccm.201908-1581ST

26.	Shorr AF, Owens RC, Jr. Guidelines and quality for community-acquired pneumonia: measures from the Joint Commission and the Centers for Medicare and Medicaid Services. *American journal of health-system pharmacy*. Jun 15 2009;66(12 Suppl 4):S2–7. doi:10.2146/090087a

27.	US Centers for Medicare & Medicaid Services. CMS Consensus-Based Entity (CBE) Endorsement and Maintenance. Accessed 8/15/2025, https://mmshub.cms.gov/sites/default/files/Blueprint-CMS-CBE-Endorsement-Maintenance.pdf

28.	Gupta AB, Flanders SA, Petty LA, et al. Inappropriate Diagnosis of Pneumonia Among Hospitalized Adults. *JAMA Intern Med*. May 1 2024;184(5):548–556. doi:10.1001/jamainternmed.2024.0077

29.	Vaughn VM, Gandhi TN, Hofer TP, et al. A Statewide Collaborative Quality Initiative to Improve Antibiotic Duration and Outcomes in Patients Hospitalized With Uncomplicated Community-Acquired Pneumonia. *Clin Infect Dis*. Aug 31 2022;75(3):460–467. doi:10.1093/cid/ciab950

30.	Gupta A, Petty L, Gandhi T, et al. Overdiagnosis of urinary tract infection linked to overdiagnosis of pneumonia: a multihospital cohort study. *BMJ Qual Saf*. May 2022;31(5):383–386. doi:10.1136/bmjqs-2021-013565

31.	Vaughn VM, Gandhi TN, Chopra V, et al. Antibiotic Overuse After Hospital Discharge: A Multi-hospital Cohort Study. *Clin Infect Dis*. Dec 6 2021;73(11):e4499–e4506. doi:10.1093/cid/ciaa1372

32.	Gandhi TN, Vaughn VM, Petty LA, et al. 2893. The Michigan Hospital Medicine Safety Consortium: Improving Patient Care by Reducing Excessive Antibiotic Use in Patients Hospitalized with Community-Acquired Pneumonia. *Open Forum Infectious Diseases*. 2019;6(Supplement_2):S80–S81. doi:10.1093/ofid/ofz359.171

33.	White AT, Vaughn V, Petty LA, et al. Development of patient safety measures to identify inappropriate diagnosis of common infections. *Clin Infect Dis*. Jan 31 2024;doi:10.1093/cid/ciae044

34.	Partnership for Quality Measurement. Inappropriate diagnosis of community-acquired pneumonia (CAP) in hospitalized medical patients, CBE ID 3671. Accessed 9/24/2024, https://p4qm.org/measures/3671

35.	Partnership for Quality Measurement. Inappropriate diagnosis of urinary tract infection (UTI) in hospitalized medical patients, CBE ID 3690. Accessed 7/28/25, https://p4qm.org/measures/3690

36.	Partnership for Quality Measurement. Percent of hospitalized pneumonia patients with chest imaging confirmation, CBE ID 4440e. Accessed 9/25/2024, https://p4qm.org/measures/4440e

37.	Yi SH, Hatfield KM, Baggs J, et al. Duration of Antibiotic Use Among Adults With Uncomplicated Community-Acquired Pneumonia Requiring Hospitalization in the United States. *Clin Infect Dis*. Apr 17 2018;66(9):1333–1341. doi:10.1093/cid/cix986

38.	Madaras-Kelly KJ, Burk M, Caplinger C, et al. Total duration of antimicrobial therapy in veterans hospitalized with uncomplicated pneumonia: Results of a national medication utilization evaluation. *Journal of hospital medicine*. Dec 2016;11(12):832–839. doi:10.1002/jhm.2648

39.	Gandhi T, Petty L, Vaughn V, et al. Risk Factors and outcomes associated with inappropriate empiric broad-spectrum antibiotic use in hospitalized patients with community-acquired pneumonia. *Antimicrobial Stewardship & Healthcare Epidemiology*. 2023;3(S2):s31–s32. doi:10.1017/ash.2023.258

40.	Adams JL. *The Reliability of Provider Profiling: A Tutorial*. RAND Corporation; 2009.

41.	Qian ET, Casey JD, Wright A, et al. Cefepime vs Piperacillin-Tazobactam in Adults Hospitalized With Acute Infection: The ACORN Randomized Clinical Trial. *Jama*. Oct 24 2023;330(16):1557–1567. doi:10.1001/jama.2023.20583

42.	Graber CJ, Jones MM, Chou AF, et al. Association of Inpatient Antimicrobial Utilization Measures with Antimicrobial Stewardship Activities and Facility Characteristics of Veterans Affairs Medical Centers. *Journal of hospital medicine*. May 2017;12(5):301–309. doi:10.12788/jhm.2730

43.	Kelly AA, Jones MM, Echevarria KL, et al. A Report of the Efforts of the Veterans Health Administration National Antimicrobial Stewardship Initiative. *Infect Control Hosp Epidemiol*. May 2017;38(5):513–520. doi:10.1017/ice.2016.328

44.	Goetz MB, Willson T, Rubin MA, Stevens VW, Graber CJ. Antimicrobial use before and during COVID-19: data from 108 Veterans Affairs medical centers. *Antimicrobial Stewardship & Healthcare Epidemiology*. 2024;4(1):e109. e109. doi:10.1017/ash.2024.352

45.	Hayes BH, Haberling DL, Kennedy JL, Varma JK, Fry AM, Vora NM. Burden of Pneumonia-Associated Hospitalizations: United States, 2001-2014. *Chest*. Feb 2018;153(2):427–437. doi:10.1016/j.chest.2017.09.041

46.	Ramirez JA, Wiemken TL, Peyrani P, et al. Adults Hospitalized With Pneumonia in the United States: Incidence, Epidemiology, and Mortality. *Clin Infect Dis*. Nov 13 2017;65(11):1806–1812. doi:10.1093/cid/cix647

47.	Burton DC, Flannery B, Bennett NM, et al. Socioeconomic and racial/ethnic disparities in the incidence of bacteremic pneumonia among US adults. *Am J Public Health*. Oct 2010;100(10):1904–11. doi:10.2105/ajph.2009.181313

48.	Altawalbeh SM, Wateska AR, Nowalk MP, et al. Societal Cost of Racial Pneumococcal Disease Disparities in US Adults Aged 50 Years or Older. *Appl Health Econ Health Policy*. Jan 2024;22(1):61–71. doi:10.1007/s40258-023-00854-0

49.	Nowalk MP, Wateska AR, Lin CJ, et al. Racial Disparities in Adult Pneumococcal Vaccination Indications and Pneumococcal Hospitalizations in the U.S. *J Natl Med Assoc*. Oct 2019;111(5):540–545. doi:10.1016/j.jnma.2019.04.011

50.	Hausmann LR, Ibrahim SA, Mehrotra A, et al. Racial and ethnic disparities in pneumonia treatment and mortality. *Med Care*. Sep 2009;47(9):1009–17. doi:10.1097/MLR.0b013e3181a80fdc

51.	Wiemken TL, Carrico RM, Furmanek SP, et al. Socioeconomic Position and the Incidence, Severity, and Clinical Outcomes of Hospitalized Patients With Community-Acquired Pneumonia. *Public Health Rep*. May/Jun 2020;135(3):364–371. doi:10.1177/0033354920912717

52.	Downing NS, Wang C, Gupta A, et al. Association of Racial and Socioeconomic Disparities With Outcomes Among Patients Hospitalized With Acute Myocardial Infarction, Heart Failure, and Pneumonia: An Analysis of Within- and Between-Hospital Variation. *JAMA Netw Open*. Sep 7 2018;1(5):e182044. doi:10.1001/jamanetworkopen.2018.2044
